# Prediction and control of fracture paths in disordered architected materials using graph neural networks

**DOI:** 10.1038/s44172-023-00085-0

**Published:** 2023-06-02

**Authors:** Konstantinos Karapiperis, Dennis M. Kochmann

**Affiliations:** Mechanics & Materials Lab, Department of Mechanical and Process Engineering, ETH Zurich, 8092 Zurich, Switzerland

**Keywords:** Mechanical engineering, Theory and computation, Mechanical properties

## Abstract

Architected materials typically rely on regular periodic patterns to achieve improved mechanical properties such as stiffness or fracture toughness. Here we introduce a class of irregular cellular materials with engineered topological and geometrical disorder, which represents a shift from conventional designs. We first develop a graph learning model for predicting the fracture path in these architected materials. The model employs a graph convolution for spatial message passing and a gated recurrent unit architecture for temporal dependence. Once trained on data gleaned from experimentally validated elastoplastic beam finite element analyses, the learned model produces accurate predictions overcoming the need for expensive finite element calculations. We finally leverage the trained model in combination with a downstream optimization scheme to generate optimal architectures that maximize the crack path length and, hence, the associated fracture energy.

## Abstract (web summary)

Karapiperis and Kochmann develop a graph neural network-based model to predict the propagation of cracks in disordered cellular materials. The approach is then applied in the design of architected structures with optimized fracture properties.

## Introduction

Predicting the propagation of cracks in cellular and lattice materials is generally considered a challenging task, especially in the presence of disorder^1^. Yet, understanding the process of crack growth is important in many contexts, such as in creating new tougher materials or in controlling the propagation of cracks away from potentially critical structural elements^2^. In fact, with the manufacturing-enabled rise of architected materials made of metals, polymers, and ceramics^3–5^, the study of their fracture properties has gained additional attention.

Despite significant theoretical progress, recent studies have outlined fundamental challenges. Concepts traditionally borrowed from linear elastic fracture mechanics (LEFM), such as the stress intensity factor and corresponding fracture toughness^6^, have been found insufficient to characterize fracture in architected materials^7,8^. In the presence of ductility, characterizing the crack tip field^9^ presents additional challenges given the extent of the plastic/process zone and its dependence on the unit cell topology^10,11^. Moreover, while most prior works have focused on periodic cellular materials, little do we know about the influence of aperiodicity, for example, through the influence of imperfections^12,13^, irregularity^1,14^, and finite size^8,15^, on fracture behavior.

Physics-based models of fracture, although predictive, are often computationally expensive^11,16–19^, which hinders their application in understanding the above effects, let alone exploiting them in material design and optimization^20^. On the other hand, machine learning has proved successful in learning complex mechanical phenomena^21^, and it has recently also been applied to the problem of fracture, predominantly for the purpose of building predictive surrogate models. For example, recent studies^22,23^ predicted fracture paths in brittle materials. Yet, those were limited to predicting whether or not preexisting cracks coalescence—a classification task. The latter was augmented by a prediction of the propagating crack tip position between coalesced cracks in ref. ^24^. Convolutional neural networks to predict crack paths were developed in refs. ^25,26^ and, more recently, have been combined with long short-term memory (LSTM) architectures in refs. ^18,27,28^. Similarly, machine learning methods have been developed to learn fracture paths in atomistic systems^29,30^ as well as to study the influence of defects on the failure of graphene^31^ and silica glass^32^. Note that most of these approaches depend on convolutions, which are restricted to a Cartesian representation of the domain of interest, and they do not directly apply to cellular materials.

Going beyond fracture prediction, a few efforts have been reported that aimed at engineering the fracture path in materials by exploiting, e.g., hierarchy^33^, multiple phases^34,35^, and grain boundaries^36^. In terms of design approaches, genetic algorithms have been developed for polycrystalline solids^37^, while greedy methods have been utilized in the design of pixel-based materials^38^. Most studies related to the design of periodic cellular materials have been restricted to heuristic or bioinspired approaches, e.g., by introducing a regular pattern of topological alterations^2^, creating interpenetrating phases^35^, or by engineering the strut thickness in honeycombs and creating softer struts through which the cracks can propagate^39^. The use of optimization-based techniques to control the fracture path in cellular materials or the explicit incorporation of disorder as a design tool have both remained relatively unexplored.

In this work, we develop a graph neural network-based surrogate model to predict the propagation of cracks in cellular materials. The model works by learning a mapping between the local geometry/topology near the crack tip and the incremental crack advance. We demonstrate this methodology on Voronoi architectures ranging from near-perfectly ordered to highly disordered. A comprehensive dataset of these architectures is generated by sampling from a carefully constructed statistical ensemble. We develop and validate—using simple tests of 3D-printed specimens—a reduced-order elastoplastic beam model, which serves as the basis for creating a large dataset of mode-I fracture simulations. The latter is used to train a machine learning model whose architecture combines graph convolutions and gated recurrent units. Once its prediction accuracy is established, the machine-learned model is leveraged in a downstream optimization task to maximize the length of the fracture path and the associated fracture energy.

## Results

### Disordered cellular solids

**Fig. 1:**
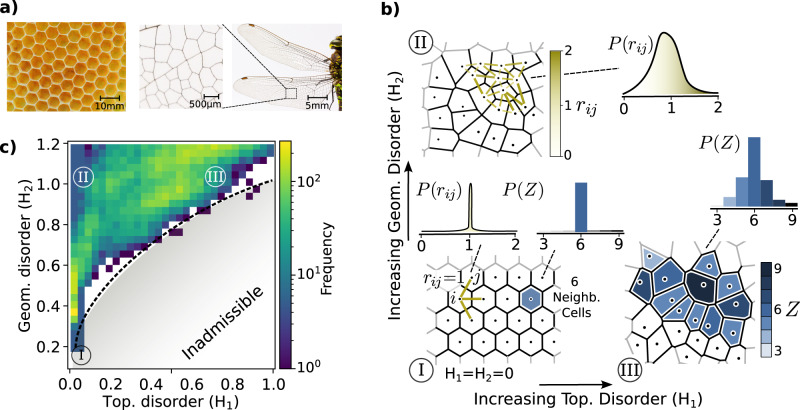
Design space of disordered cellular materials. **a** Examples of a regular and an irregular cellular architecture in nature. Left: bee honeycomb (Source: Ante Hamersmit, CC0 License), right: dragonfly wing (Source: Pixabay, CC0 License). **b** I: Perfectly regular honeycomb. II: Interpretation of geometrical disorder in terms of the distribution of internuclei distances. III: Interpretation of topological disorder in terms of the distribution of local coordination numbers (number of cell neighbors). **c** Frequency histogram in disorder space for our full dataset.

Toward a versatile albeit simple design of disorder cellular solids, we focus on Voronoi tessellations in 2D, i.e., on truss networks generated by partitioning space into cells, each surrounding a nucleus. Such tessellations are prevalent in nature (Fig. 1a). The cells’ edges define sets of points that are equidistant to the two closest nuclei (Fig. 1b). To extend the classical construction of Voronoi networks (see, e.g., ref. ^40^) into a more powerful and descriptive approach, we consider a statistical ensemble of those networks with a controllable range of geometrical and topological disorder. We define the topological disorder *H*_1_ as the variance of the local coordination number *Z* (number of neighbors of a Voronoi cell), i.e., *H*_1_ = 〈*Z*^2^〉 − 〈*Z*〉^2^. An interpretation is given in Fig. 1b, which shows cells colored according to their coordination number as well as their statistical distribution in the whole domain. Similarly, we define the geometrical disorder *H*_2_ as the variance of the internuclei distance, i.e., ${H}_{2}=\langle {r}_{ij}^{2}\rangle -{\langle {r}_{ij}\rangle }^{2}$, where *i*, *j* denote neighboring nuclei (Fig. 1b). We associate *H*_1_, *H*_2_ = 0 with a regular hexagonal honeycomb. Note that this is a design choice; we could alternatively consider, e.g., a perfect square tessellation. Choosing increasing values of *H*_1_, *H*_2_ leads to increasingly disordered designs. As outlined in “Methods”, we proceed to generate a statistical ensemble, sampling from which results in a dataset of roughly 50,000 architectures, each comprised of *N* = 162 nuclei and ranging from perfectly ordered (*H*_1_, *H*_2_ → 0) to highly disordered (*H*_1_, *H*_2_ > 1). The histogram of the corresponding topological and geometrical disorder in the dataset is shown in Fig. 1c, illustrating the number of realizations sampled for each combination. Note that due to the interplay of topological and geometrical disorder, one cannot populate the entire *H*_1_-*H*_2_ space. Instead, for a given *H*_2_, a certain maximum value of *H*_1_ can be accommodated. This notion is reflected in the inadmissible region shown in Fig. 1c.

### Mechanical properties

**Fig. 2:**
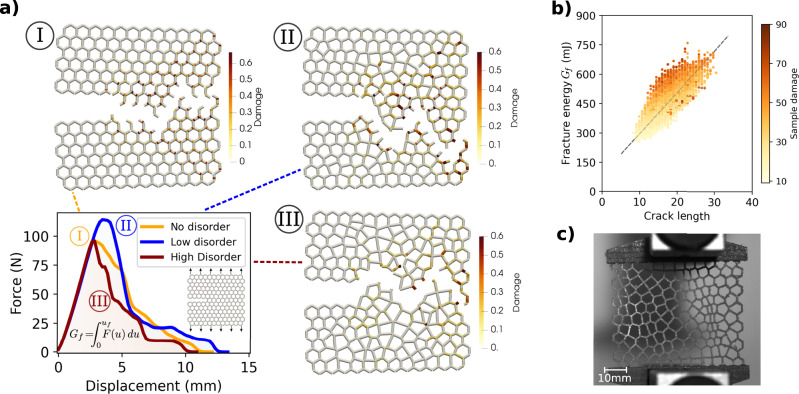
Fracture mechanics of disordered cellular materials. **a** Force-displacement curves and corresponding fracture patterns, resulting from simulations of three specimens, using the elastoplastic beam finite element model and different levels of disorder: I) perfectly regular, II) low disorder, III) high disorder. **b** Fracture energy vs. crack length (number of broken edges). **c** Experimental setup for validation, using an unnotched irregular 3D-printed specimen.

We consider the canonical mode-I fracture problem^7,29,41^, as shown in Fig. 2a, which involves a sample loaded vertically in tension. This is the simplest setting that abides by the ASTM standards^42^ and is used here to reveal the physics of the fracture process. A notch is placed at the left side of a rectangular sample (amounting to 25% of its width) by removing any intersecting struts. To minimize boundary effects, we keep one layer of cells around the exterior boundary at their regular positions (effectively reducing the disorder to zero in this thin zone). The upper and lower boundaries are displaced vertically at a constant rate, leading to the opening of the crack.

Simulations of such systems typically either rely on fully resolved finite element (FEM) calculations^39^, which results in prohibitive computational costs for producing a sufficiently large dataset for learning, or simple strut failure criteria based on maximum pointwise stress or strain^35^, which may result in an oversimplification of the mechanics. To mitigate these issues, we develop an accurate albeit efficient reduced-order corotational beam model that captures the essential underlying mechanics. The base material is described by a 1D model featuring rate-dependent damage and plastic hardening. The model is calibrated and validated against tensile experiments of samples that are additively manufactured out of a mixture of acrylic photopolymer and thermoplastic elastomer using a Connex polyjet printer (Fig. 2c). The details of the material model can be found in “Methods”, while its calibration and validation is presented in Supplementary Note 2.

In the case of quasi-regular networks (*H*_2_ < 0.5), we observe oscillatory fracture patterns (e.g., Fig. 2a - I/II), verifying similar observations in earlier studies^14^, with cracks propagating continuously from the crack tip. As disorder increases, cracks may, in some cases, appear a few cells away from the crack tip and later coalesce, indicating a form of crack bridging^1^, while, in other cases, smaller cracks disconnected from the main crack may form. The force-displacement curves in Fig. 2a reveal that some degree of disorder may be beneficial for obtaining higher fracture energy ${G}_{f}=\int\nolimits_{0}^{{u}_{f}}F(u)du$ (i.e., the area under the curve), where *u* and *F* denote the boundary displacement and net force, respectively. Interestingly, the data from ref. ^1^ also hint at the possibility of a beneficial effect of moderate disorder, but the authors did not investigate this in detail. Figure 2b plots *G*_*f*_ for all networks in the dataset. As reported in prior studies of other materials (e.g., ref. ^43^), we find that the value of *G*_*f*_ correlates with the length of the crack path (i.e., the number of broken edges). This is expected since most of the absorbed energy is derived from the release of elastic energy previously stored in beams that will eventually fracture. Several other mechanisms, including the accumulation of damage and plasticity in beams that will eventually not reach the point of fracture, are also at play but do not significantly affect the value of *G*_*f*_.

### Machine learning model

**Fig. 3:**
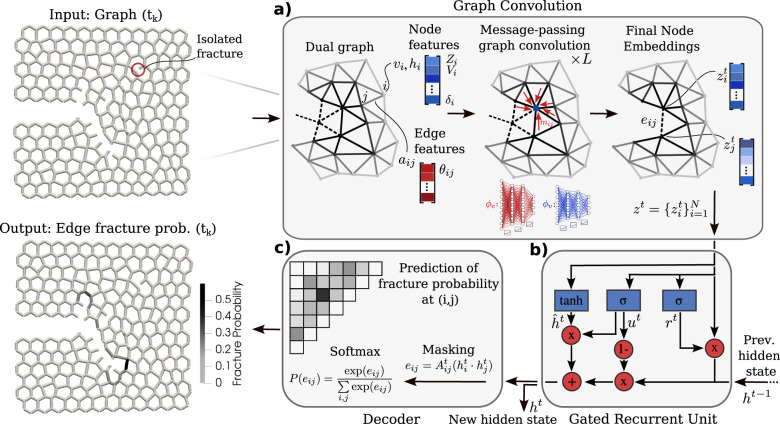
Architecture of the spatiotemporal graph neural network. **a** Message-passing convolution, **b** Gated recurrent Unit, **c** Inner-product decoder. For better illustration, only the graph near the crack tip is shown.

For each of the simulated samples, we save the updated graph connectivity every time a beam is broken, ignoring any isolated broken beams away from the crack path. This results in a sequence of as many graphs as the number of (non-isolated) broken edges. This set of sequences of graphs constitutes our training data. As shown in Fig. 3, the machine learning model takes as input the current configuration at step *t* (i.e., *t* broken edges), along with a hidden state encapsulating the history from previous steps. It outputs a fracture probability for all intact beams (network edges), along with a new hidden state. At any given time, the crack path is simply the set of edges that represent the difference in connectivity between the original graph (*t* = 0) and the current graph (*t*). The model features three fundamental components. The spatial component (Fig. 3a) accounts for the effect of the neighborhood of a crack tip on the stress concentration/redistribution and, hence, the crack advance. It is implemented as a custom message-passing graph convolution^44^. The temporal component (Fig. 3b) incorporates any pertinent history effects of the process and is furnished by a gated recurrent unit^45^. Finally, the inner-product decoder (Fig. 3c) is the mathematical machinery used to translate the previous components into a probability of edge breaking. We briefly discuss each of the components in the following, yet a more detailed formulation can be found in Supplementary Note 3.

The purpose of the spatial message-passing convolution is to learn the relation between the topological and geometrical features encoded in the nodes and edges and the probability of an intact edge breaking next. Figure 3a shows the details of the graph convolution employed, which is based on the general framework of ref. ^44^. We operate on the dual graph (Voronoi nuclei represent nodes of the dual graph, and edges between Voronoi cells correspond to the relevant dual edges^46^), on which we define appropriate nodal features **v**_*i*_ (cell volume, anisotropy vector, circularity, coordination number, geometrical order parameter) and edge features **a**_*ij*_ (edge orientation, edge length). We refer to Supplementary Note 3 for a detailed mathematical definition of each feature. The nodal features are updated by a message-passing operation as follows: for each node *i*, we form a message **m**_*ij*_ originating from a neighboring node *j* by passing the nodes’ features **v**_*i*_, **v**_*j*_, their positions **x**_*i*_, **x**_*j*_, and the edge features **a**_*ij*_ through a learnable function, here a fully connected neural network. This operation is summarized as **m**_*ij*_ = *ϕ*_*e*_(**v**_*i*_, **v**_*j*_, **x**_*i*_, **x**_*j*_, **a**_*ij*_). The messages from the set of neighbors of *i*, i.e., ${{{{{{{\mathcal{N}}}}}}}}(i)$ are aggregated through a simple summation ${{{{{{{{\bf{m}}}}}}}}}_{i}={\sum }_{j\in {{{{{{{\mathcal{N}}}}}}}}(i)}{{{{{{{{\bf{m}}}}}}}}}_{ij}$. A second neural network takes as input this aggregated message as well as the original node feature **v**_*i*_ to produce the new node feature ${{{{{{{{\bf{v}}}}}}}}}_{i}^{{\prime} }={\phi }_{v}({{{{{{{{\bf{v}}}}}}}}}_{i},{{{{{{{{\bf{m}}}}}}}}}_{i})$ for node *i*. This whole operation constitutes one convolutional layer and results in updating all nodal features by accumulating topological and geometrical information from neighbors one hop away. We find that six spatial convolutions produce the most accurate results. This mirrors the idea of a fracture process zone of a similar topological distance where stress redistribution occurs^1^. Upon applying these convolutions, each node *i* now holds the final nodal features or embeddings ${{{{{{{\bf{z}}}}}}}}={\{{{{{{{{{\bf{z}}}}}}}}}_{i}\}}_{i\le N}$.

Since the fracture process can include history effects due to damage and plasticity, we also incorporate a temporal aspect into the model. We introduce a gated recurrent unit (GRU)^45^, which is a modern and more efficient version of the long short-term memory network (LSTM)^47^, a widely used recurrent neural network architecture. The underlying idea is to combine the node embeddings **z**^*t*^ derived from the spatial convolution at a given step *t*, as described above, with a hidden embedding **h**^*t*−1^ of the same dimensionality, which is continuously updated and incorporates the memory of all previous steps. As shown in Fig. 3b, the GRU incorporates an update gate **u**_*t*_ = *σ*(*W*_*u*_[**z**^*t*^, **h**^*t*−1^] + **b**_*u*_) used to retain useful information from the node embeddings of the previous step, and a reset gate **r**_*t*_ = *σ*(*W*_*r*_[**z**^*t*^, **h**^*t*−1^] + **b**_*r*_) used to forget the embedding information of the previous step, which is no longer relevant for future predictions. Note that *σ*, *W*, and *b* denote the sigmoid activation function, weights, and biases of these gates, respectively, while **h**^0^ is a collection of zero vectors (embeddings), indicating the absence of memory at the first step. With the help of a few additional numerical manipulations, the next hidden state **h**^*t*^ is computed, as described in detail in Supplementary Note 3. Overall, the GRU computes the state at step *t* through the hidden state at step *t* − 1 and the current state at step *t*. Note that similar ideas of combing graph convolutions with recurrent architectures have been recently used in traffic forecasting^48^.

To compute the likeliest edge to break at step *t*, we first pass the current hidden state **h**^*t*^ through an inner-product decoder^49^. This produces a matrix with components ${{{{{{{{\bf{h}}}}}}}}}_{i}^{t}\cdot {{{{{{{{\bf{h}}}}}}}}}_{j}^{t}$. Clearly, only existing edges are eligible to be broken; hence, we use the current adjacency matrix as a mask (via element-wise multiplication) to compute ${e}_{ij}^{t}={A}_{ij}^{t}({{{{{{{{\bf{h}}}}}}}}}_{i}^{t}\cdot {{{{{{{{\bf{h}}}}}}}}}_{j}^{t})$, where no summation over identical indices is implied. For the sake of efficiency, we further assume that the crack advances by breakage of edges adjacent to the cells of the current path, although, as discussed above, in some occasions, edges may break up to a couple of cells away and then coalesce. In these cases, the order in which network edges are assumed to fracture deviates slightly from the ground truth. Finally, a softmax layer translates the value ${e}_{ij}^{t}$ corresponding to each edge into a probability of breaking, $p({e}_{ij}^{t})$. The training of the model amounts to minimizing the binary cross entropy ${{{{{{{\mathcal{L}}}}}}}}=-1/N\mathop{\sum }\nolimits_{i = 1}^{n}{\hat{p}}_{ij}^{t}\log [p({e}_{ij}^{t})]+(1-{\hat{p}}_{ij}^{t})\cdot \log [1-p({e}_{ij}^{t})]$, where ${\hat{p}}_{ij}^{t}=1$ for the true edge that breaks at step *t* and 0 for all other edges. Additional details regarding hyperparameters, data augmentation, and training parameters are discussed in “Methods”.

### Prediction accuracy

**Fig. 4:**
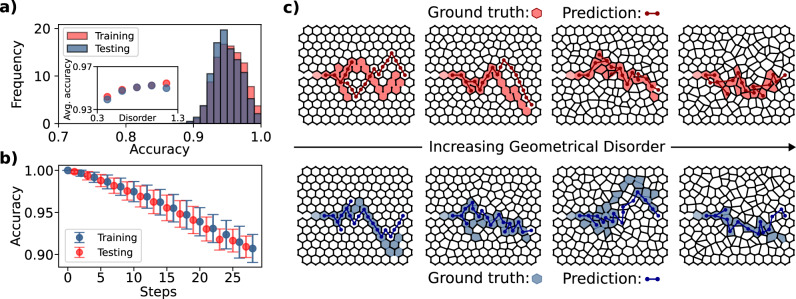
Trained graph neural network. **a** Accuracy histogram for predictions on the training (*n* = 45,000 samples) and test dataset (*n* = 5000 samples) for the entire fracture path. Inset: Average accuracy vs. geometrical disorder. **b** Evolution of the accuracy of the prediction on the training and test dataset as a function of the number of steps (i.e., number of broken beams). Error bars represent ±1 standard deviation. **c** Randomly sampled architectures for a range of different values of geometrical disorder (upper row: training set, lower row: test set). Ground truth (light filled nuclei—broken primal edges) is compared with model predictions (dark dual edges).

Once trained, the model predicts the crack advance at arbitrary lengths through an incremental approach, similar to ref. ^18^. To evaluate the accuracy of the trained model, we plot the ratio of correctly predicted edges (e.g., edges that were predicted to break and indeed broke) over the sum of correctly and incorrectly predicted edges for the entire fracture path (Fig. 4a). The average accuracy is similar for the training and test sets (both above 90%), indicating that no overfitting has taken place. Restricting this calculation to the first *t* steps (i.e., the first *t* broken beams of a fracture path) and repeating it for increasing *t* allows us to compute the timewise evolution of the accuracy. Figure 4b shows the statistics (mean ± std. deviation) of this *t*-step accuracy across multiple predictions in the training and test sets. As expected, the accuracy decreases with increasing steps (i.e., increasing the number of broken beams) as it becomes more and more likely for the prediction to diverge from the correct fracture path. This represents a limitation of the current model in evaluating very long paths, which could be addressed with a more sophisticated temporal model. The inset of Fig. 6a shows the accuracy as a function of the geometrical disorder *H*_2_, which highlights that the accuracy decreases as regularity increases. This is anticipated since, in the limit of perfect honeycombs, fracture may propagate upward or downward with equal probability due to symmetry. This is also evident in Fig. 4c, which compares model predictions and ground truth fracture patterns for samples randomly drawn from the training and test set at different values of disorder. For example, in the upper left plot (corresponding to low disorder), the model predicts a fracture pattern that is a mirror of the ground truth. As disorder increases, predictions generally improve. It is finally worth noting that the use of the softmax layer for the prediction of the next edge to break naturally provides an estimate of the confidence of the model’s prediction. The closer to 1 the value of the evaluated edge after the softmax layer, the more accurate the individual prediction is for a given increment.

### Speed and memory efficiency

**Fig. 5:**
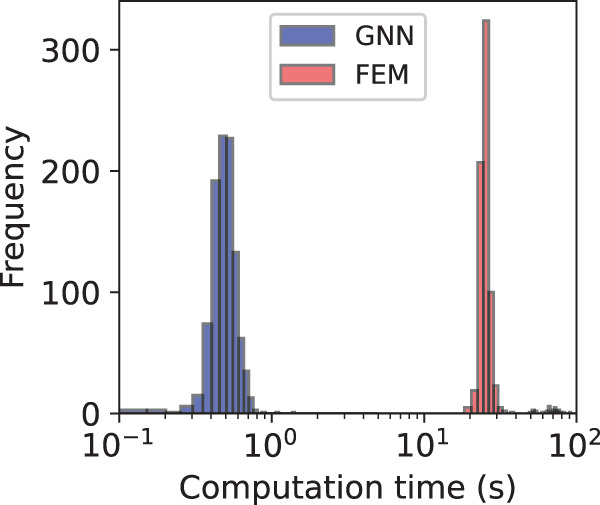
Speed performance of the framework. Comparison between the time in seconds for predicting the fracture paths using FEM and the proposed graph framework (*n* = 1000 independent experiments).

To gauge the performance of the framework in terms of speed, we measure the prediction time for 1000 different networks, sampled across the entire dataset, and compute the mean and standard deviation. For the same networks, we also calculate the statistics of the computation time of the highly optimized FEM solver. As shown in Fig. 5, the proposed graph learning framework delivers almost 2 orders of magnitude faster predictions than FEM. It is worth noting that this comes with a substantial memory footprint during training, which stems from expensive neighborhood aggregation operations^50,51^. Fortunately, the nature of the problem allows us to keep the memory consumption constant despite increasing network size. This is due to the concept of a K-dominant region around the crack tip^1,6^, outside of which the stresses decay rapidly. This implies that the prediction of the next edge to fail only depends on the subgraph covering a finite region around the instantaneous crack tip, while the rest of the graph can be ignored, allowing us to efficiently handle large graphs. If, for 3D extensions of the framework, that finite subgraph still leads to substantial memory consumption, then solutions can be sought in a combination of techniques, including using compressed^52^ or historical embeddings^53^, decoupling neighborhood aggregation from prediction^54,55^, adopting subgraph^56,57^ training, and using more scalable architectures relying on reversible connections^58^ or anisotropic convolutions^59^. All the above adaptations have the potential to reduce the framework’s memory footprint.

### Optimizing the fracture toughness

We leverage our trained model toward designing cellular architectures with maximal fracture toughness. The latter is interpreted here as the fracture energy, which is widely used in similar studies^39,60,61^. This interpretation of toughness overcomes insufficiencies of the LEFM interpretation and the need to consider lattices where the region of *K*_*I*_ dominance is much larger than the cell size. Moreover, we can take advantage of the observed correlation between the fracture energy and the length of the crack path (Fig. 2b) and tackle the proxy problem of maximizing the latter.

**Fig. 6:**
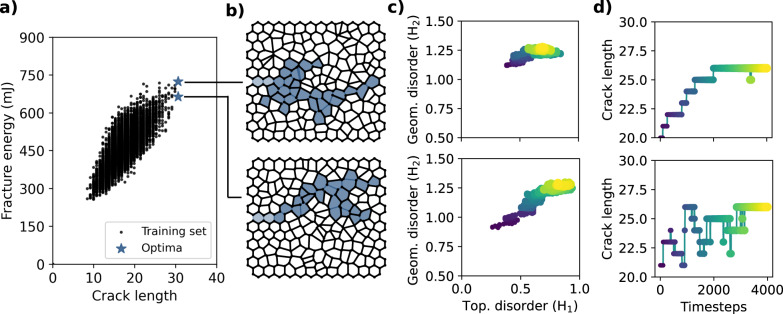
Sample optimization. **a** Fracture energy vs. topological crack path length for the training set (⋅) and two examples of optimal points obtained through MCMC (⋆) along with **b** the corresponding optimizers (Voronoi networks). **c** Evolution of the topological and geometrical disorder for each of the networks throughout the optimization. **d** Evolution of the predicted crack length throughout the optimization. The color map indicates optimization steps from beginning () to end ().

We particularly consider two optimization problems. First, we focus on discovering specific Voronoi networks (i.e., spatial arrangements of nuclei) that exhibit the longest fracture paths. Since we can rapidly evaluate fracture patterns using the trained model, we opt for a sampling-based optimization. To do so, we sequentially perturb an initial arrangement of nuclei using a simple Markov Chain Monte Carlo (MCMC) scheme. A given perturbation is accepted with a probability that depends on the effected change of length of the fracture path, as predicted by the trained model. The algorithm is given in more detail in “Methods”. We carry out this optimization procedure starting from 20 initial arrangements and report the best optima. These are shown in Fig. 6a in terms of the topological crack length and the associated fracture energy, evaluated upon the end of optimization by FEM analysis. The corresponding optimizers (Voronoi networks) and the fracture patterns are shown in Fig. 6b. Interestingly, the optimal networks exhibit relatively diffuse crack patterns, which include crack branching. Finally, we report the evolution of the predicted crack length and disorder metrics throughout the optimization process in Fig. 6c and d, respectively.

**Fig. 7:**
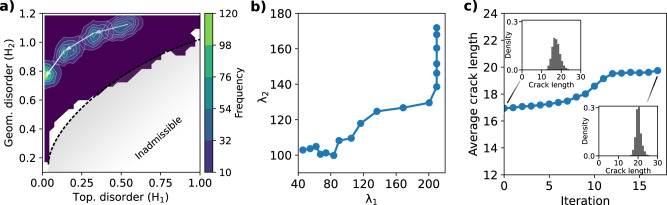
Ensemble optimization. **a** Evolution of the probability density of topological and geometrical disorder during optimization. **b** Evolution of the generators (Lagrange multipliers) controlling the Voronoi networks. **c** Average crack length vs. iteration number.

Second, ensemble optimization allows us to look for the generators of the ensemble (*λ*_*i*_, see “Methods”) that produce networks exhibiting, on average maximal crack path lengths and hence maximal fracture energies. To this end, we adapt the statistical physics-inspired design engine set forth in ref. ^62^ to our problem. The major advantage of this method is that it incorporates knowledge about the configuration space. We start with an initial value of the generators and incrementally update them in an optimal manner. The exact algorithm is detailed in “Methods”. Figure 7a shows the evolution of the probability density of the geometrical and topological disorder of the networks until convergence. Note that the ensemble optimal networks lie in a region of the disorder space characterized by higher regularity (lower *H*_1_, *H*_2_ values) compared to the previous individually optimized networks. In particular, we find that the optimal topological disorder approaches zero, i.e., topological defects, on average, do not increase the fracture energy. Finally, Fig. 7b shows the evolution of *λ*_*i*_, while Fig. 7c shows the average crack length of the evolving ensemble until convergence. We are able to achieve a 20% increase in this average length.

## Discussion

We have developed a design methodology for irregular architected cellular materials, which represents a paradigm shift from conventional periodic designs. By utilizing two effective statistical metrics that represent topological and geometrical disorders, we have introduced a new approach for parameterizing the design of such materials. By moving these statistical descriptors from the dual to the primal space, the design space can be expanded beyond Voronoi-based architectures. We have focused on the challenging task of optimizing their fracture energy, with a wide range of applications in materials science and engineering, including the design of tough structural materials and aerospace components^63^, biomedical implants^64^, and electronics^65^. Our machine learning framework, which allows for the rapid prediction of crack patterns in these cellular media, leverages their natural description as graphs and results in a learned mapping from the space of local topological and geometrical features at the vicinity of a crack tip to its incremental crack advance. The trained model relies on a number of assumptions, including mode-I loading as well as a specific notch configuration and beam description. However, the framework itself is applicable under a wider range of conditions (e.g., mode-II loading or Timoshenko beams) as long as there exists a pronounced notch leading to stress concentrations. It will be interesting to investigate its generalization capabilities under these modified assumptions. We have also shown how the framework can be used for the design of optimal architectures that maximize fracture energy. Unlike in prior approaches, disorder in cellular materials is explicitly used to optimize nonlinear properties beyond the simple engineering of defects in an otherwise perfectly periodic architecture. Finally, the framework can prove useful in designing the fracture path, e.g., steering it away from specific parts of the domain, or it may be utilized toward optimizing combined mechanical properties beyond fracture. Overall, this study can pave the way for a new class of architected disordered materials, especially upon its extension to 3D.

## Methods

### Generation of random architectures

The disordered architectures in this study have been generated as follows. We consider a statistical ensemble ${{{{{{{{\mathcal{G}}}}}}}}}_{n}$ of Voronoi networks composed of *n* nuclei. The ensemble is constructed by maximizing the entropy subject to a normalization constraint and two additional constraints which serve to control the degree of disorder. This is mathematically stated as

1 $$\mathop{\max }\limits_{P}- 	\mathop{\sum}\limits_{G\in {{{{{{{{\mathcal{G}}}}}}}}}_{n}}P(G)\log P(G),\\ \,{{\mbox{s.t.}}}\,\quad 	\mathop{\sum}\limits_{G}P(G){H}_{i}(G)={\hat{H}}_{i}(G),\quad i=1,2,\\ 	\mathop{\sum}\limits_{G}P(G)=1,$$

where *G* denotes the Voronoi network or graph, *P*(*G*) is the probability of its occurrence in the ensemble, *H*_1_ = 〈*Z*^2^〉 − 〈*Z*〉^2^ is a measure of the topological disorder of a network (i.e., the variance of the local coordination number *Z*), and ${H}_{2}=\langle {r}_{ij}^{2}\rangle -{\langle {r}_{ij}\rangle }^{2}$ is a measure of its geometrical disorder (i.e., the variance of the internuclei distance *r*_*ij*_). The solution of Eq. (1) gives the well-known Boltzmann distribution:

2 $$P(G)=\frac{1}{{{{{{{{\mathcal{Z}}}}}}}}}\exp \Big(-\mathop{\sum}\limits_{i}{\lambda }_{i}{H}_{i}(G)\Big),$$

where ${{{{{{{\mathcal{Z}}}}}}}}$ denotes the partition function. In practice, instead of enforcing specific values of ${\hat{H}}_{i}$ directly, we consider a range of corresponding *λ*_*i*_, then sample from the corresponding distributions above using MCMC^66^, and inspect the resulting *H*_*i*_. This construction provides greater control than the typical construction of random Voronoi lattices based only on the minimum internuclei distance (see, e.g., ref. ^40^). Note that sampling one Voronoi network requires 5000–10,000 MCMC iterations, depending on the *λ*_*i*_-values (i.e., the degree of disorder). During each iteration, the nuclei positions are perturbed, a new Voronoi tesselation is computed, the resulting “energy” of the system, ∑*λ*_*i*_*H*_*i*_, is calculated, and the perturbed positions are accepted or rejected based on standard MCMC criteria. The entire process takes, on average, 120s in our unoptimized Python code running on an Intel i7 1.30GHz processor.

### Fracture simulations

We adopt an Euler–Bernoulli corotational formulation for the beam kinematics in our cellular architectures^67^. We postulate the existence of an energy potential ${{{{{{{\mathcal{W}}}}}}}}(\varepsilon ,{\varepsilon }_{p})$ as well as a dissipation potential ${\phi }^{* }({\dot{\varepsilon }}_{p})$, where *ε* is the longitudinal strain and *ε*_*p*_ is the plastic longitudinal strain. Restricting our attention to slender beams, we adopt a fiber-type formulation^68^, in which the strain contributions are uncoupled. In order to capture the mechanical behavior seen in experiments, we adopt the following form for the potentials, based on a plastic strain driven damage-law (*d*), a power law-based plastic hardening energy, and a rate-dependent dissipation potential:

3 $${{{{{{{\mathcal{W}}}}}}}}=\frac{1}{2}(1-d)E{(\varepsilon -{\varepsilon }^{p})}^{2}+\frac{C}{\kappa +1}| {\varepsilon }^{p}{| }^{\kappa +1},$$

4 $$d=1-\exp (-s| {\varepsilon }^{p}| ),$$

5 $${\phi }^{* }({\dot{\varepsilon }}^{p})={\sigma }_{0}| {\dot{\varepsilon }}^{p}| +{\tau }_{0}\frac{{\dot{\varepsilon }}_{0}}{m+1}{\left\vert \frac{{\dot{\varepsilon }}^{p}}{{\dot{\varepsilon }}_{0}}\right\vert }^{m+1},$$

depending on Young’s modulus *E*, hardening parameters *C* and *κ*, damage parameter *s*, and strength parameters *σ*_0_, *τ*_0_, and *m*. Additional details about the model formulation, its calibration and validation against experiments are given in Supplementary Notes 1 and 2.

### Graph neural network model

Our graph neural network depicted in Fig. 3a includes fully connected edge and node networks, each having three layers and feature rectified linear units as activation layers. The overall graph network has six layers, a hidden nodal dimension of 256, and a latent nodal dimension of 32. A relevant hyperparameter study can be found in Supplementary Note 4. Residual layers are introduced in the graph network to avoid the known problem of oversmoothing^69^. Dropout layers with a dropout probability of 0.4 are used to avoid overfitting. For the purpose of data augmentation, the domain and the associated fracture pattern are flipped vertically, effectively doubling the size of the dataset. During training, we monitor the model performance on the test set, comprised of 10% of the total data, after each epoch. We employ the ADAM optimizer^70^, with a learning rate of 0.00005. We perform early stopping when the testing accuracy metric stops decreasing for six consecutive epochs to avoid overfitting. The model is implemented in the open-source library Pytorch-Geometric^71^.

### Fracture path optimization—sample

We adopt a sequential optimization algorithm furnished by a Markov Chain Monte Carlo approach. The algorithm takes the following steps: (1) take *N* nuclei and distribute them either randomly on the domain or such that they correspond to a desired initial value of topological and geometrical disorder. (2) Construct the Voronoi graph and compute associated node/edge features. (3) Evaluate the trained machine learning model to predict the fracture path length *L*. (4) Move the nucleus of a randomly chosen Voronoi cell by a small random displacement. (5) Recompute (locally) the Voronoi tesselation, update the graph features, and reevaluate the fracture path length ${L}^{{\prime} }$. If ${L}^{{\prime} } > \, L$, accept the move. If ${L}^{{\prime} }\le L$ only accept with a probability ${e}^{-({L}^{{\prime} }-L)/T}$, where *T* is a temperature-like hyperparameter, otherwise do not move the nucleus. (6) Check for convergence in the overall improvement of *L*. (7) Go to step (4).

### Fracture path optimization—ensemble

For the ensemble average optimization, we adopt a statistical physics-inspired optimization scheme. The fundamental idea is to compute the evolution of the Lagrange multipliers *λ*_*i*_ toward the direction of increasing the probability of finding the system in states with above-average values of a desired average quantity. This quantity here is the length of the crack path, *L*(*G*), given a Voronoi graph *G*. Following ref. ^62^, we desire

6 $$\dot{P}(G| {\lambda }_{i})=P(G| {\lambda }_{i})[L(G)-\langle L(G)\rangle ],$$

where 〈⋅〉 denotes averaging over the configuration space weighted by ${P}_{{\lambda }_{i}}$. This translates into

7 $${\dot{\lambda }}_{i}={\langle {\partial }_{{\lambda }_{i}}\log (P){\partial }_{{\lambda }_{j}}\log (P)\rangle }^{-1}\langle [L(G)-\langle L(G)\rangle ]{\partial }_{{\lambda }_{j}}\log (P)\rangle .$$

Using Eq. (2), the above equation further transforms into

8 $${\dot{\lambda }}_{i}={\langle {H}_{i}(G){H}_{j}(G)\rangle }^{-1}\langle L(G){H}_{j}(G)\rangle .$$

Averaging over the configuration space is computed by sampling 2000 configurations in parallel. For each, the crack path can be rapidly evaluated using the trained graph model.

### Supplementary information

Supplementary Information

## Data Availability

The source data for all figures, as well as the data required to train the machine learning model, are available in the ETH Research Collection (10.3929/ethz-b-000608722)^72^.

## Code availability

The code for the machine learning model developed in this study is available from GitHub (https://github.com/kkarapiperis/gnn-fracture).
