# Supplementary material for: Prediction and control of fracture paths in disordered architected materials using graph neural networks
Source: Commun Eng. 2023 Jun 2;2:32. doi: 10.1038/s44172-023-00085-0 (PMC10956016; doi:10.1038/s44172-023-00085-0)
Supplement: Supplementary file 1 — Supplementary Information [file 44172_2023_85_MOESM1_ESM.pdf]

Prediction and control of fracture paths in disordered architected materials using graph neural networks

Sumplemental Material

Konstantinos Karapiperis, Dennis M. Kochmann

Supplementary Note 1. Beam model formulation

For all fracture simulations of the cellular architectures, we adopt a reduced-order corotational beam formulation. Fig. 1 a) shows a schematic of the adopted corotational Euler-Bernoulli beam kinematics, which is based on [4]. Restricting our attention to the two-dimensional problem, the longitudinal strain at any point (x, y) in the local coordinate system aligned with the neutral axis of a beam is given by

$$\varepsilon = \varepsilon_{xx}(x, y, z) = \varepsilon_{\text{ax.}}(x) - y\,\kappa(x) \tag{1}$$

where $\varepsilon_{\text{ax.}}$ is the axial strain along the centerline, and κ is the local curvature due to bending about the $\boldsymbol{e}_y$-axis.

We consider a variational formulation [3], involving the effective incremental potential at pseudo-time t_{k+1}:

$$\mathcal{F}_{\varepsilon^{p,k}}(\varepsilon^{k+1}, \varepsilon^{p,k+1}) = \mathcal{W}(\varepsilon^{k+1}, \varepsilon^{p,k+1}) + \Delta t\ \phi^* \left(\frac{\varepsilon^{p,k+1} - \varepsilon^{p,k}}{\Delta t} \right), \tag{2}$$

whereby we assume the following internal energy density and dissipation potentials:

$$\mathcal{W} = \frac{1}{2}(1-d)E(\varepsilon - \varepsilon^p)^2 + \frac{C}{\kappa+1}|\varepsilon^p|^{\kappa+1} \tag{3}$$

$$d = 1 - \exp(-s|\varepsilon^p|) \tag{4}$$

$$\phi^*(\dot{\varepsilon}^p) = \sigma_0|\dot{\varepsilon}^p| + \tau_0 \frac{\dot{\varepsilon}_0}{m+1}\left|\frac{\dot{\varepsilon}^p}{\dot{\varepsilon}_0}\right|^{m+1} \tag{5}$$

dependent on Young's modulus E, hardening parameters C and γ, damage parameter s, strength parameters σ_0, τ_0, and m, and the plastic strain ε^p.

The solution to the boundary value problem is the minimizer of the effective potential energy functional

$$\Pi_{\boldsymbol{\varepsilon}^{p,k}}[\tilde{\mathbf{u}}^{k+1}, \boldsymbol{\varepsilon}^{p,k+1}] = \int_{\mathcal{B}} \mathcal{F}_{\boldsymbol{\varepsilon}^{p,k}}(\boldsymbol{\varepsilon}^{k+1}(\tilde{\mathbf{u}}^{k+1}), \boldsymbol{\varepsilon}^{p,k+1}), \tag{6}$$

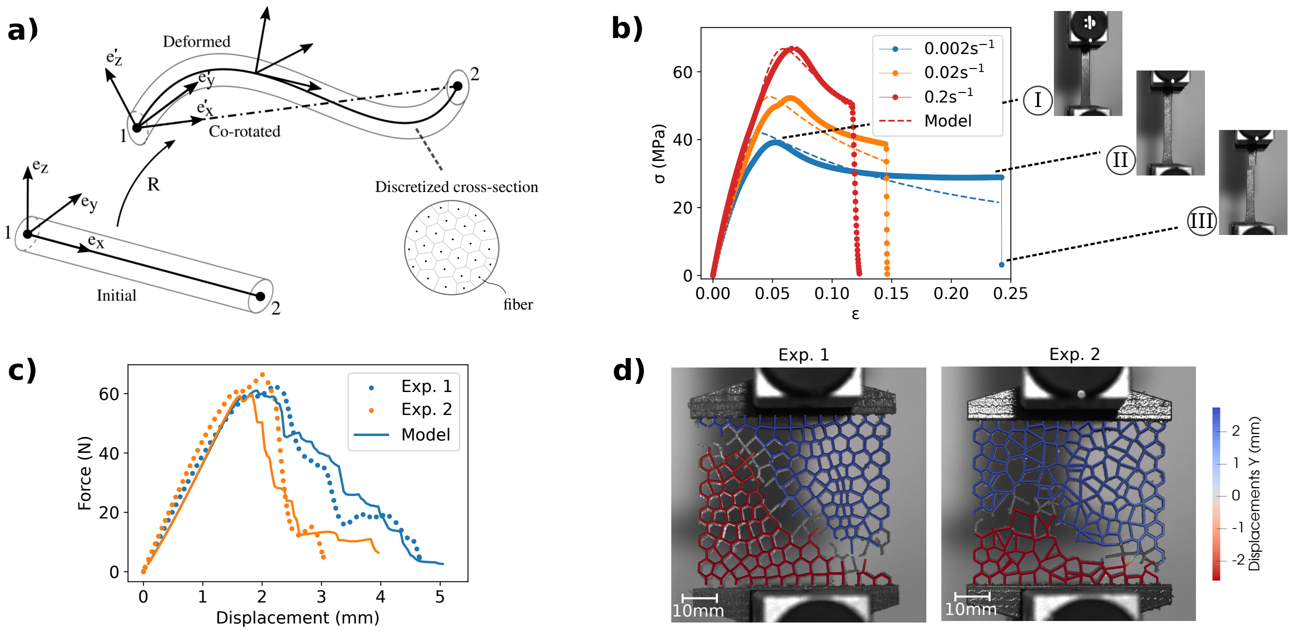

Supplementary Figure 1: **Beam model formulation, calibration and experimental validation**. **a)** Corotational kinematics of the adopted Euler-Bernoulli beam model, based on individual 1D fibers making up the cross-section. **b)** Calibration of the material model at different rates. **c)** Experimental vs. simulated force-displacement curves for the two tensile validation experiments. **d)** Final fracture patterns from the simulations (colored) overlaid on top of the experimental ones.

where $\tilde{\mathbf{u}} = [\mathbf{u}, \boldsymbol{\theta}]$ is the vector of displacements and rotational degrees of freedom, $\mathcal{B}$ is the domain of interest, and the subscripts $\boldsymbol{\varepsilon}^{p,k}$ denote the dependence of the potentials on the internal variables at the previous time t^k. Each strut is discretized into beam elements, for which we adopt the usual linear interpolation for the displacement along the local element axis, and cubic interpolation for the displacements perpendicular to it [4]. In integrating the resulting equations, we use Gauss-Lobatto integration along the beam length to better account for the influence of the nodes, and material point integration on the cross-section of the beam (Fig. 1 a). The latter furnishes an interpretation of the beam element as a collection of independent fibers [5]. The model is implemented in the open source-computational mechanics C++ library ae108 (www.ae108.ethz.ch).

Supplementary Note 2. Beam model calibration and validation

The model is calibrated against tensile experiments of ASTM dogbone samples [2], which are additively manufactured out of a mixture of acrylic photopolymer (VeroWhite) and thermoplastic elastomer (TPE) using a Connex polyjet printer. The samples are tested on an Instron Universal testing machine. Three dogbones using variable deformation rates are used, in order to capture the relevant viscoplastic effects. The result of the calibration procedure is shown in Fig. 1 b), showing that the assumed model formulation can faithfully capture the nonlinear rate-dependent response and the accumulation of damage until fracture.

For validation, two cellular samples of variable disorder and size 52.5mm $\times$ 60mm are manufactured out of the same base material, with struts of rectangular cross-section and in-plane width of 0.5mm. These are tested under the same tensile loading conditions with a rate of 333.3 mm/min. Quasistatic simulations using the calibrated values of the model are carried out, and the results are compared to the experiments. Fig. 1 c) shows that the calibrated model can satisfactorily capture the experimental force-displacement response. The model's fidelity is further verified by its ability to capture the final fracture patterns, as shown in Fig. 1 d).

Supplementary Note 3. Machine learning model formulation

We describe in detail the mathematical formulation of the spatiotemporal graph neural network shown in Fig. 3.

Graph representation and node features

In our dual graph representation, each node i (among N total nodes) corresponds to a Voronoi cell, and is endowed with the following nodal features:

$$\mathbf{v}_i = [V_i, \mathbf{x}_i - \mathbf{b}_i, c_i, Z_i, \delta_i], \tag{7}$$

where V_i is the Voronoi cell's volume, x_i is the position of the cell's nucleus, $\mathbf{b}_i$ is the cell's barycenter (such that $\mathbf{x}_i - \mathbf{b}_i$ defines its anisotropy), c_i is the cell's circularity defined as $1/(4\pi$ area/perimeter$^2)$, Z_i is its coordination number defined as the number of neighboring cells, and δ_i is a geometrical order parameter defined as the sum of distances of neighboring nuclei. Additionally, we associate each edge connecting nodes i, j with an edge feature vector

$$\mathbf{a}_{ij} = [\theta_{ij}, l_{ij}], \tag{8}$$

where θ_{ij} is the angle and l_{ij} the length of the edge.

Spatial Message Passing

A *three-step* message passing convolution is used to update the node features v_i, which involves:

i) the formation of a message m_{ij} for each neighbor $j \in \mathcal{N}(i)$ (i-neighborhood) by a nonlinear transformation (via a neural network ϕ_e) of the original features and positions of nodes i, j and the features of the edge connecting them:

$$\mathbf{m}_{ij} = \phi_e(\mathbf{v}_i, \mathbf{v}_j, \mathbf{x}_i, \mathbf{x}_j, \mathbf{a}_{ij}), \tag{9}$$

ii) the aggregation of the message to neighbors through a simple summation:

$$\mathbf{m}_i = \sum_{j \in \mathcal{N}(i)} \mathbf{m}_{ij}, \tag{10}$$

and iii) the nonlinear transformation (via a neural network ϕ_v) of the original node embedding and the message $\mathbf{m}_i$:

$$\mathbf{v}'_i = \phi_v(\mathbf{v}_i, \mathbf{m}_i). \tag{11}$$

Altogether, this leads to

$$\mathbf{v}_i' = \phi_v \left(\mathbf{v}_i, \sum_{j \in \mathcal{N}(i)} \phi_e(\mathbf{v}_i, \mathbf{v}_j, \mathbf{x}_i, \mathbf{x}_j, \mathbf{a}_{ij}) \right). \tag{12}$$

This constitutes one convolution layer, and results in the updated node embeddings $\mathbf{v}_i'$ above for all nodes i in the graph. Repeating this process results in $\mathbf{v}_i''$, and so forth. Overall, stacking n convolutional layers results in the final node embeddings, which, following standard conventions, we denote as $\mathbf{z}_i$, i.e.,

$$\mathbf{z}_i \equiv \mathbf{v}_i^{(n)} = \phi_v \left(\mathbf{v}_i^{(n-1)}, \sum_{j \in \mathcal{N}(i)} \phi_e(\mathbf{v}_i^{(n-1)}, \mathbf{v}_j^{(n-1)}, \mathbf{x}_i, \mathbf{x}_j, \mathbf{a}_{ij}) \right). \tag{13}$$

Note that $n = 6$ layers are used in our framework.

Gated recurrent unit

The collection of nodal embeddings $\mathbf{z}^t = \{\mathbf{z}_i^t\}_{i \leq N}$ is calculated at each step t based on the current state of the graph (i.e., where edges that correspond to broken beams up to this step have been removed). However, these embeddings do not incorporate direct information of the history up to step t. Therefore, we compute a so-called hidden embedding $\mathbf{h}^t$, which starts as a vector of zeros and is continuously updated by keeping the relevant information of each $\mathbf{z}^t$. This is achieved using an architecture termed *gated recurrent unit* [1]. Its mathematical formulation at the first step is:

$$\mathbf{u}^1 = \sigma(W_u[\mathbf{z}^1, \cancelto{0}{\mathbf{h}^0}] + \mathbf{b}_u), \tag{14}$$

$$\mathbf{r}^1 = \sigma(W_r[\mathbf{z}^1, \cancelto{0}{\mathbf{h}^0}] + \mathbf{b}_r), \tag{15}$$

$$\hat{\mathbf{h}}^1 = \phi_h(W_h[\mathbf{z}^1, \mathbf{r}^1 \odot \cancelto{0}{\mathbf{h}^0}] + \mathbf{b}_h), \tag{16}$$

$$\mathbf{h}^1 = (1 - \mathbf{z}^1) \odot \cancelto{0}{\mathbf{h}^0} + \mathbf{z}^1 \odot \hat{\mathbf{h}}^1, \tag{17}$$

where σ, W_u, W_r, and $\mathbf{b}_u, \mathbf{b}_r$ denote the sigmoid activation function, weights, and biases, respectively, and $\odot$ is the Hadamard product. Similarly, at any next step we combine $\mathbf{z}^t$ and $\mathbf{h}^{t-1}$ to compute $\mathbf{h}^t$ via

$$\mathbf{u}^t = \sigma(W_u[\mathbf{z}^t, \mathbf{h}^{t-1}] + \mathbf{b}_u), \tag{18}$$

$$\mathbf{r}^t = \sigma(W_r[\mathbf{z}^t, \mathbf{h}^{t-1}] + \mathbf{b}_r), \tag{19}$$

$$\hat{\mathbf{h}}^t = \phi_h(W_h[\mathbf{z}^t, \mathbf{r}^t \odot \mathbf{h}^{t-1}] + \mathbf{b}_h), \tag{20}$$

$$\mathbf{h}^t = (1 - \mathbf{z}^t) \odot \mathbf{h}^{t-1} + \mathbf{z}^t \odot \hat{\mathbf{h}}^t. \tag{21}$$

This formulation of the gated recurrent unit is shown schematically in Fig. 3b).

Inner product decoder

An inner product decoder is used to convert the hidden state $\mathbf{h}^t = \{\mathbf{h}_i^t\}_{i \leq N}$ to a probability of each edge being removed (i.e., beam breaking). It is mathematically formulated as an $N \times N$ matrix whose components are given by

$$e_{ij}^t = A_{ij}^t(\mathbf{h}_i^t \cdot \mathbf{h}_j^t), \tag{22}$$

where A_{ij}^t is the (i, j)-entry of the adjacency matrix at time t (such that e_{ij}^t is zero for non-existent edges), $(\cdot)$ denotes a dot product, and no summation over identical indices is implied. As discused in the main text, for the sake of efficiency we additionally set A_{ij}^t to zero for any edge (i, j) that is not in the immediate neighborhood of the nodes indicating the position of the crack tip up to step t. This allows us to disregard any isolated beam failures. Finally, a softmax layer translates the value e_{ij}^t corresponding to each edge into a probability of breaking, $p(e_{ij}^t)$ according to

$$p(e_{ij}^t) = \frac{\exp(e_{ij}^t)}{\sum_{k,l} \exp(e_{kl}^t)}. \tag{23}$$

Loss function

We use a standard binary cross entropy loss function $\mathcal{L} = -\frac{1}{N}\sum_{i=1}^{n} \hat{p}_{ij}^t \, \log[p(e_{ij}^t)] + (1 - \hat{p}_{ij}^t) \log[1 - p(e_{ij}^t)]$, where $\hat{p}_{ij}^t = 1$ for the true edge that breaks at step t and 0 for all other edges.

Supplementary Note 4. Machine learning model hyperparameter study

A study was carried out to assess the effect of hyperparameters on the accuracy of the spatiotemporal graph neural network, focusing on i) the number of convolutional layers, ii) the hidden nodal dimension, iii) the latent nodal dimension, and iv) the learning rate employed during training. The results are shown in Fig. S2. We find that the most influential parameters are the number of layers and the hidden nodal dimensions. The model's test accuracy increases with increasing number of layers, yet for more than 6 layers the increase is less pronounced probably as a result of oversmoothing. Similarly, the higher the hidden nodal dimension, the higher the accuracy of the model, which becomes more expressive. A learning rate in the range $5 \cdot 10^{-5} - 10^{-4}$ is optimal, as it is sufficiently small to avoid exploding gradients, while being sufficiently large to converge within a reasonable training time.

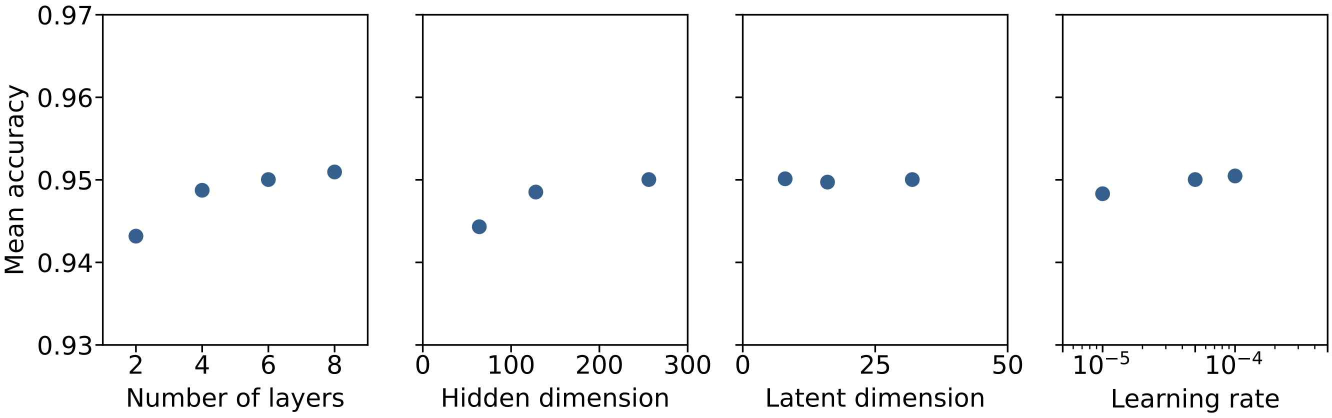

Supplementary Figure 2: **Effect of GNN hyperparameters (number of convolutional layers, hidden dimension, latent dimension, learning rate) on the model's test accuracy.**

Supplementary References

[1] Cho, K. et al. "On the properties of neural machine translation: Encoder-decoder approaches". In: *arXiv preprint arXiv:1409.1259* (2014).

[2] International, ASTM. "Standard test methods for tensile properties of plastics". In: *West Conshohocken, PA* (2014).

[3] Ortiz, M. and Stainier, L. “The variational formulation of viscoplastic constitutive updates”. In: *Computer methods in applied mechanics and engineering* 171.3-4 (1999), pp. 419–444.

[4] Phlipot, G. P. and Kochmann, D. M. “A quasicontinuum theory for the nonlinear mechanical response of general periodic truss lattices”. In: *Journal of the Mechanics and Physics of Solids* 124 (2019), pp. 758–780.

[5] Spacone, E., Filippou, F. C., and Taucer, F. F. “Fibre beam–column model for non-linear analysis of R/C frames: Part I. Formulation”. In: *Earthquake Engineering & Structural Dynamics* 25.7 (1996), pp. 711–725.
